# Increased activity of DRD1-MSNs in dorsolateral striatum underlies *Cry1Δ11* mutation-induced repetitive behaviors

**DOI:** 10.1038/s41398-026-04219-8

**Published:** 2026-07-13

**Authors:** Xiran Liu, Dengfeng Liu, Bingyu Long, Yichun Zhang, Fan Xia, Jia-Da Li, Suixin Deng

**Affiliations:** 1 Furong Laboratory, Center for Medical Genetics, School of Life Sciences, Central South University, Changsha, Hunan P. R. China; 2 MOE Key Laboratory of Rare Pediatric Diseases, Changsha, Hunan P. R. China; 3 Hunan Key Laboratory of Animal Models for Human Diseases, Changsha, Hunan P. R. China; 4 Hunan Key Laboratory of Medical Genetics, Changsha, Hunan P. R. China; 5 Hunan International Scientific and Technological Cooperation Base of Animal Models for Human Diseases, Changsha, Hunan P. R. China

**Keywords:** Molecular neuroscience, Medical genetics

## Abstract

Repetitive behaviors are classically associated with autism spectrum disorder and obsessive compulsive disorder, but also occur prominently in attention-deficit/hyperactivity disorder (ADHD), yet the underlying mechanisms remain poorly understood. Our recent work identified ADHD-like behaviors in *Cry1Δ11* mice, in which a mutation in a core circadian gene *Cry1* produces a *CRY1Δ11* protein that fails to inhibit the Gαs subunit, leading to hyperactive signaling of dopamine D1 receptor (DRD1). Although this dysregulation was initially reported in the ventral striatum, we hypothesized that similar mechanisms might be present in the dorsal striatum, a brain region critically involved in the generation of repetitive behaviors and densely populated by DRD1-expressing medium spiny neurons (MSNs). Here, we demonstrate that *Cry1Δ11* mice exhibit robust repetitive behaviors, including excessive self-grooming and stereotyped rearing, which are associated with increased activity of DRD1-MSNs in the dorsolateral striatum. Chemogenetic manipulation further revealed that activation of these neurons induces excessive self-grooming, whereas their inhibition reduces such behavior, indicating bidirectional control over repetitive action. Critically, systemic administration of the DRD1 antagonist SCH23390 fully rescued both neuronal hyperactivity and behavioral abnormalities in mutant mice. Together, our findings establish a direct mechanistic link among a core circadian gene mutation, striatal dopaminergic hyperactivity, and repetitive behaviors, thereby identifying aberrant DRD1 signaling in the dorsolateral striatum as a promising target for therapeutic intervention.

## Introduction

Repetitive behaviors constitute a broad class of actions characterized by stereotyped movements, insistence on sameness, and restricted interests. Although traditionally associated with autism spectrum disorder (ASD) and obsessive compulsive disorder (OCD), accumulating clinical evidence indicates that such behaviors are also prevalent in individuals with attention-deficit/hyperactivity disorder (ADHD) [1–3]. In children with ADHD, repetitive behaviors are frequently observed [1–4], with specific symptoms showing notable overlap: hyperactivity correlates with stereotyped motor behaviors, impulsivity with restricted preferences, and impulsive speech with repetitive verbal output [5]. Although this co-occurrence has been attributed to shared deficits in executive dysfunction, including impairments in behavioral inhibition and cognitive generativity [6, 7], the underlying molecular mechanisms remain poorly understood.

In contrast, growing evidence suggests an association between ADHD and circadian/sleep dysregulation. Children with ADHD exhibit significantly higher rates of sleep disturbances compared to neurotypical peers, including prolonged sleep onset latency, increased daytime sleepiness, and a pronounced evening chronotype [8]. At the physiological level, altered melatonin secretion rhythms and delayed circadian phase markers have been consistently reported in ADHD populations [9]. Given that circadian rhythms (endogenous approximately 24-hour oscillations governing behavior and physiology) are orchestrated by a cell-autonomous molecular clock, it is plausible that disruptions in core clock genes contribute not only to the sleep abnormalities and core neuropsychiatric features of ADHD but also to the co-occurrent repetitive behaviors.

The mammalian circadian clock operates through a transcriptional-translational negative feedback loop. Central to this mechanism are the core clock proteins BMAL1 and CLOCK, which form heterodimers that bind E-box elements to drive expression of Period (*Per1/2*) and Cryptochrome (*Cry1/2*) genes [10, 11]. The resulting PER and CRY proteins then translocate to the nucleus and inhibit BMAL1/CLOCK activity, thereby repressing their own transcription [12]. Thus, CRY proteins serve as potent transcriptional repressors and key modulators of clock precision [13].

A pivotal advance linking circadian dysfunction to neuropsychiatric traits came in 2017, when Patke et al. identified a splice-site mutation in *CRY1* (c.1657+3A>C), termed *CRY1Δ11*, in families with delayed sleep phase disorder (DSPD) [14]. This mutation disrupts the 5’ splice donor site of exon 11, leading to exon skipping and a 24-amino-acid deletion in the C-terminal tail of CRY1 protein. Subsequent phenotypic analyses revealed that carriers of *CRY1Δ11* also display elevated rates of psychiatric conditions such as ADHD [15]. Our recent works further demonstrated that *Cry1Δ11* mutant mice recapitulate multiple ADHD-like phenotypes, such as hyperactivity, impulsivity, learning deficits [16], and altered pain sensitivity [17]. Mechanistically, Zhang et al. demonstrated that wild-type (WT) CRY1 binds to the Gαs subunit and suppresses dopamine D1 receptor (DRD1) signaling [18]; our previous work revealed that the *CRY1Δ11* mutation disrupts this interaction, abolishing CRY1’s inhibitory function and resulting in hyperactive DRD1 signaling in the ventral striatum (Nucleus accumbens, NAc) [16].

Given that this molecular lesion is intrinsic to the *CRY1Δ11* protein itself and could therefore operate in any brain region expressing DRD1, we hypothesized that the same pathogenic mechanism might underlie comorbid repetitive behaviors in ADHD by driving neuronal hyperactivity in the dorsal striatum, a region densely populated with DRD1-expressing neurons and critically involved in motor stereotypies. Here, we report that *Cry1Δ11* mice exhibit robust repetitive behaviors, including excessive self-grooming and stereotyped rearing. Using chemogenetic approaches, we demonstrate that hyperactivation of DRD1-expressing neurons in the dorsolateral striatum is both necessary and sufficient to drive the repetitive behavior. Furthermore, administration of DRD1 antagonist effectively rescues both neuronal hyperactivity and repetitive abnormalities in *Cry1Δ11* mice. Together, our findings offer a novel mechanistic framework for understanding the co-occurrence of ADHD and repetitive behaviors and identifying aberrant DRD1 signaling as a promising therapeutic target.

## Results

### *Cry1Δ11* mutation promotes repetitive behaviors

**Fig. 1:**
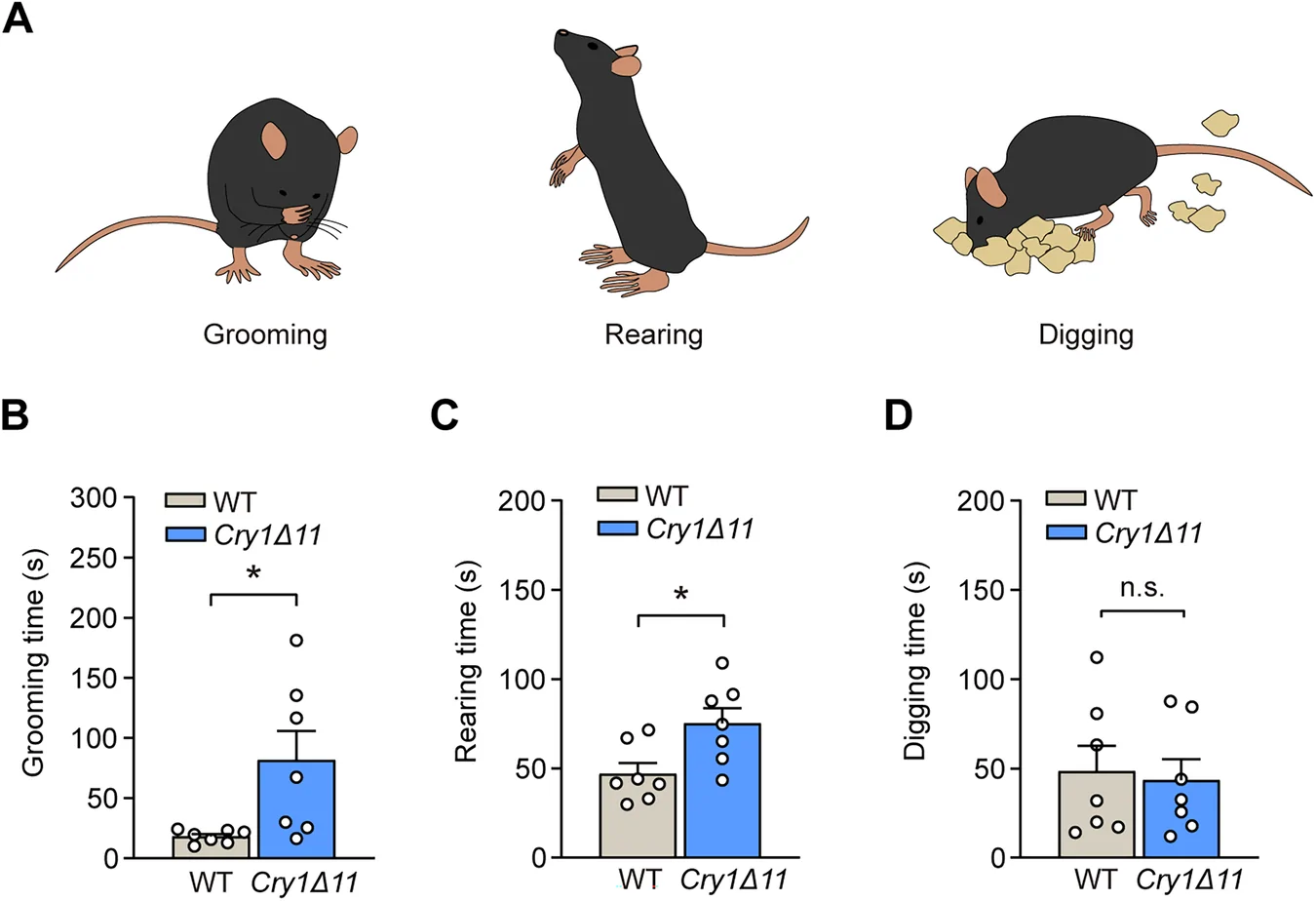
*Cry1Δ11* mice exhibit increased repetitive behaviors. **A** Schematic illustration of the repetitive behaviors assessed in the cylinder test: grooming, rearing, and digging. **B–D** Quantification of time spent engaged in grooming **(B)**, rearing **(C)**, and digging **(D)** in *Cry1Δ11* mice (n = 7) compared to wild-type (WT) littermates (n = 7). *P < 0.05, ns, not significant (P ≥ 0.05), Welch’s test for grooming and two-tailed unpaired Student’s *t* test for rearing and digging. Data are presented as mean ± SEM.

Given the frequent co-occurrence of repetitive behaviors in individuals with ADHD, we assessed repetitive behaviors-related phenotypes in *Cry1Δ11* mice using an open cylinder test (see Methods) that quantifies three stereotyped actions: self-grooming, rearing and digging [19, 20] (Fig. 1A). Based on our previous observation [16] that *Cry1Δ11* mice exhibit robust ADHD-like behaviors during zeitgeber time 14–16 (ZT14-16), all behavioral assays were conducted at this circadian phase.

In the open cylinder test, *Cry1Δ11* mice spent significantly more time engaged in self-grooming compared with WT littermates (Fig. 1B). Detailed behavioral analysis further revealed that rearing duration was markedly elevated in *Cry1Δ11* mice relative to WT controls (Fig. 1C). In contrast, no significant difference was observed in digging behavior between two genotypes (Fig. 1D). These results indicate that the *Cry1Δ11* mutation specifically enhances certain forms of repetitive behavior, particularly self-grooming and rearing.

### Hyperactivation of DRD1 signaling in the dorsolateral striatum of *Cry1Δ11* mice

Substantial evidence implicates the dorsal striatum as a critical node for the generation of repetitive behaviors, with dopaminergic system dysregulation in this region strongly linked to stereotyped actions [19, 21–25]. However, whether dorsal striatum also contributes to repetitive behavior in the context of ADHD remains unclear. Our previous work demonstrated that *Cry1Δ11* mice show hyperactive DRD1 signaling in the NAc due to loss of CRY1-mediated inhibition of the Gαs subunit, which underlies core ADHD-like phenotypes, including hyperactivity, impulsivity, and learning deficits [16]. Thus, we hypothesized that similar DRD1 hyperactivity may also occur in the dorsal striatum, which contains abundant DRD1-expressing neurons and may underlies the observed abnormalities in repetitive behaviors.

**Fig. 2:**
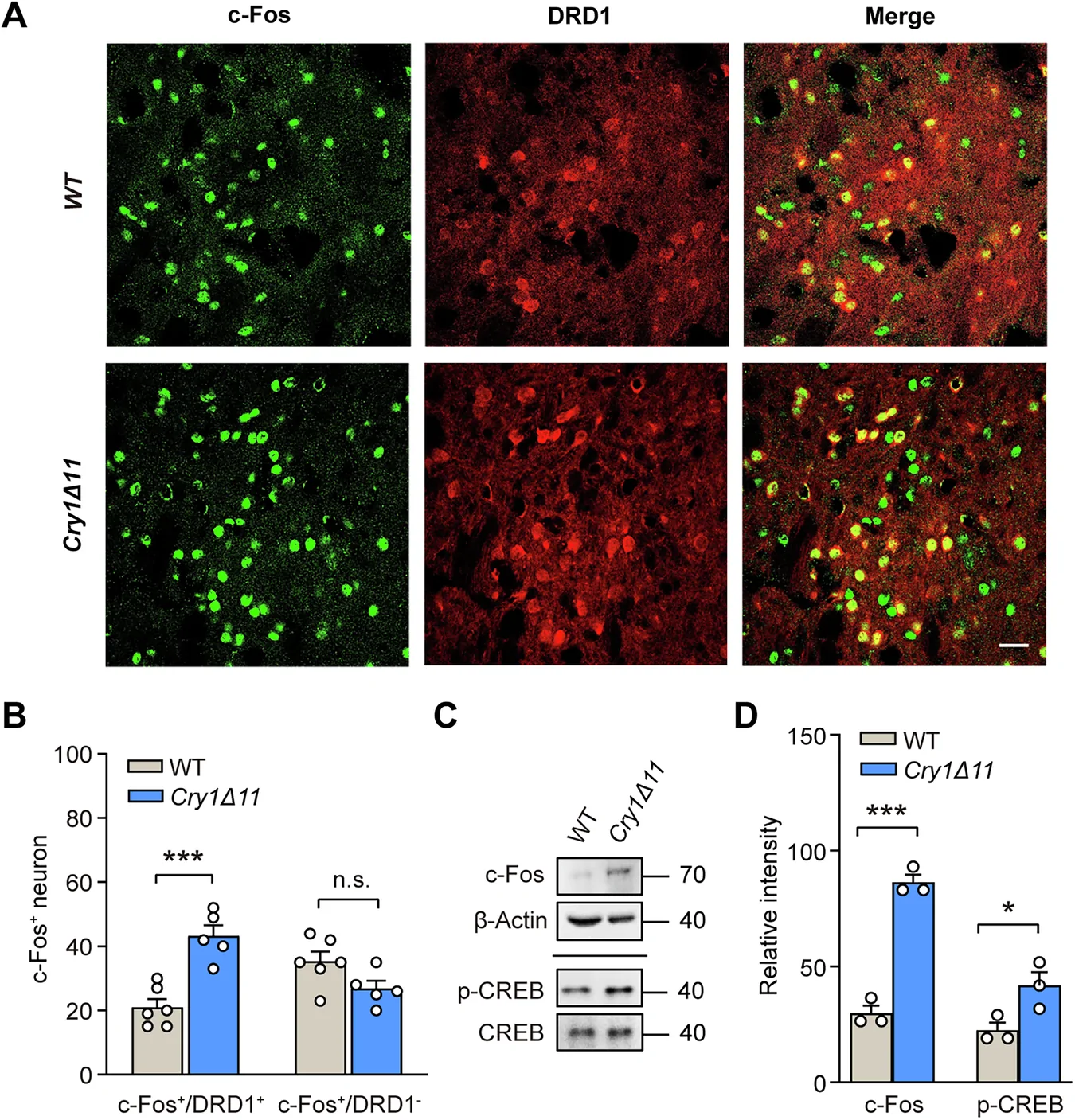
Hyperactive DRD1 signaling in the dorsal striatum of *Cry1Δ11* mice. **A** Representative immunofluorescence images showing c-Fos expression in DRD1-MSNs in the dorsal striatum of WT and *Cry1Δ11* mice. Scale bar, 10 μm. **B** Quantification of c-Fos^+^/DRD1^+^ MSNs and c-Fos^+^/DRD1^-^ neurons in the dorsal striatum. n = 6 WT versus 5 *Cry1Δ11* mice. ***P < 0.001, ns, not significant (P ≥ 0.05), two-way ANOVA followed by Bonferroni post hoc test. **C**, **D** Representative western blots **(C)** and quantification **(D)** of c-Fos and phosphorylated CREB (p-CREB) protein levels in dorsal striatum lysates from WT and *Cry1Δ11* mice. Both c-Fos and p-CREB were significantly upregulated in *Cry1Δ11* mice. n = 3 WT versus 3 *Cry1Δ11* mice. *P < 0.05, ***P < 0.001, two-tailed unpaired Student’s *t* test. Data are presented as mean ± SEM.

To assess the activation of DRD1 signaling pathway in the dorsal striatum, we examined expression of the immediate-early gene c-Fos and phosphorylated cAMP response element-binding protein (p-CREB), both established markers of neuronal activity and cAMP/PKA signaling. Immunofluorescence staining revealed significantly elevated c-Fos immunoreactivity in the dorsal striatum of *Cry1Δ11* mice compared with WT controls (Fig. 2A). Double-labeling analysis showed that the majority of c-Fos-positive cells co-expressed DRD1 [26], and the number of c-Fos⁺/DRD1⁺ neurons was markedly increased in *Cry1Δ11* mice, whereas c-Fos⁺/DRD1⁻ neurons (Putative DRD2-expressing neurons) did not differ between genotypes (Fig. 2B). Western blot analysis further confirmed that both c-Fos and p-CREB protein levels were significantly upregulated in dorsal striatum tissue from *Cry1Δ11* mice (Fig. 2C, D and Supplementary Fig. 1), mirroring the pattern previously observed in the NAc [16]. These data indicate that the cAMP-dependent DRD1 signaling cascade is enhanced in DRD1-expressing neurons of dorsal striatum in *Cry1Δ11* mice.

### Increased activity of putative DRD1-MSNs in the dorsal striatum of *Cry1Δ11* mice

**Fig. 3:**
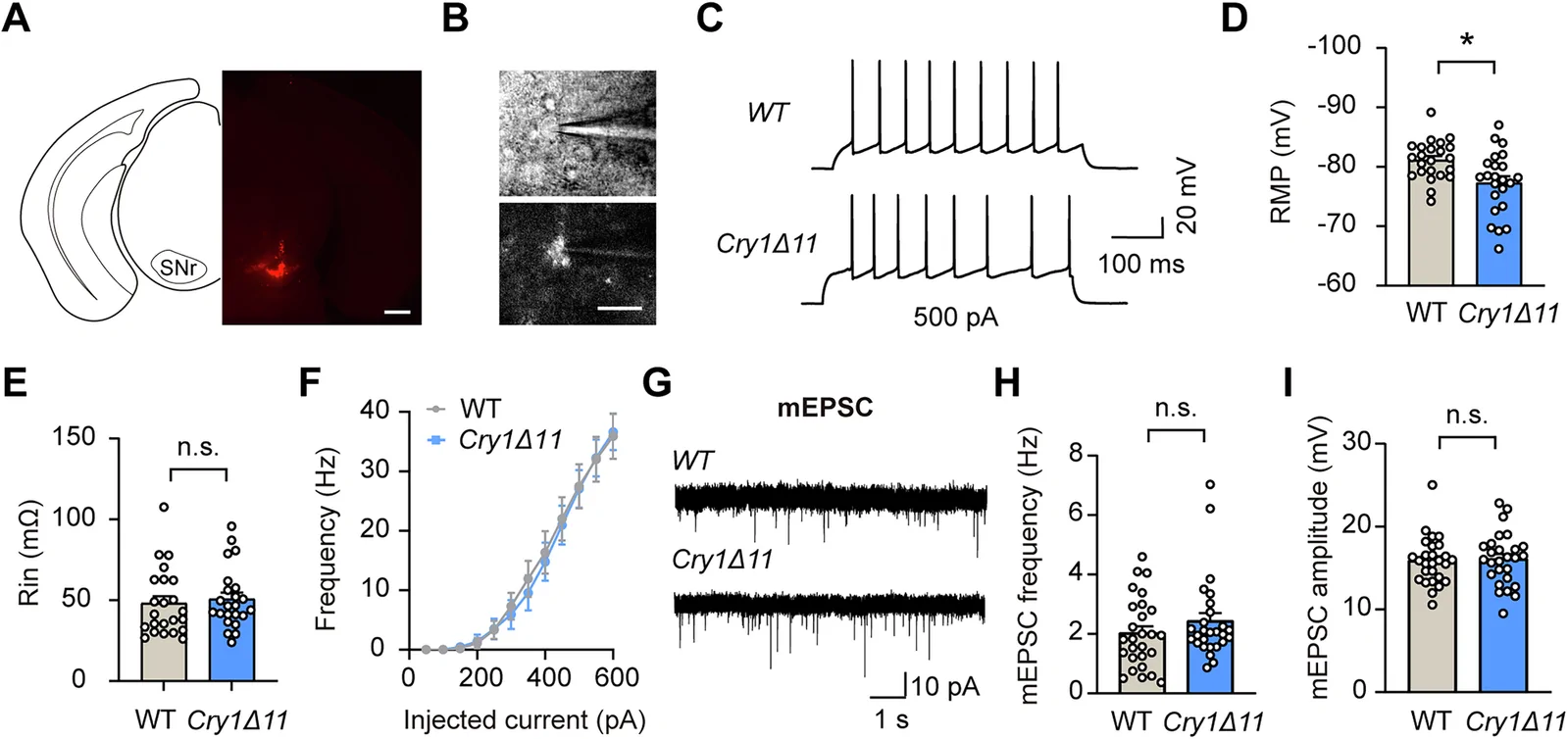
Dorsal striatal putative DRD1-MSNs exhibit increased neuronal activity in *Cry1Δ11* mice. **A** Example coronal brain section showing red retrobeads labeling in SNr. Scale bar, 500 μm. **B** Representative differential interference contrast (DIC; top) and fluorescence (bottom) images of putative DRD1-MSNs in the dorsal striatum. Scale bar, 10 μm. **C** Representative action potentials evoked by 500 pA current injections in putative DRD1-MSNs from WT and *Cry1Δ11* mice. **D**, **E** The resting membrane potential (RMP; **D**) and input resistance (Rin; **E**) of putative DRD1-MSNs from two groups. n = 23 neurons from 6 WT mice versus 23 neurons from 6 *Cry1Δ11* mice. *P < 0.05, Welch’s test. **F** Action potential firing frequency in response to a series of depolarizing current steps in putative DRD1-MSNs from two groups. **G** Sample traces of mEPSC recorded from putative DRD1-MSNs in the presence of PTX (50 μM) and TTX (1 µM) in WT (top) and *Cry1Δ11* (bottom) mice. **H**, **I** Quantification of mEPSC frequency **(H)** and amplitude **(I)** from WT and *Cry1Δ11* mice. n = 27 neurons from 6 WT mice versus 27 neurons from 7 *Cry1Δ11* mice. Mann-Whitney *U* tests for mEPSC frequency **(H)** and two-tailed unpaired Student’s *t* test for mEPSC amplitude **(I)**. ns, not significant (P ≥ 0.05). Data are presented as mean ± SEM.

To determine whether elevated DRD1 signaling in *Cry1Δ11* mice leads to increased neuronal excitability, we performed whole-cell patch-clamp recordings from direct pathway neurons in the dorsal striatum. The dorsal striatum contains two major populations of GABAergic medium spiny neurons (MSNs): DRD1-MSNs form the direct pathway, and DRD2-MSNs constitute the indirect pathway. DRD1-MSNs project directly to basal ganglia output nuclei, including the substantia nigra pars reticulata (SNr) and internal globus pallidus (GPi). To selectively target this population, we injected fluorescent retrobeads into the SNr three days before brain slice preparation, thereby labeling striatal MSNs that project directly to the SNr (Fig. 3A). These retrobead-positive cells in the dorsal striatum were thus considered putative DRD1-MSNs for subsequent electrophysiological analysis (Fig. 3B).

Recordings from these putative DRD1-MSNs in *Cry1Δ11* mice revealed significantly depolarized resting membrane potential (RMP), indicating a lower current threshold for action potential initiation (Fig. 3C, D). However, the input resistance (Rin) and the firing frequency-current (F-I) relationship did not differ between groups (Fig. 3E, F). Furthermore, both the frequency and amplitude of miniature excitatory postsynaptic currents (mEPSCs; Fig. 3G–I) and miniature inhibitory postsynaptic currents (mIPSCs; Supplementary Fig. 2) were comparable in *Cry1Δ11* mice and WT controls, suggesting that the depolarized RMP is driven by the intrinsically heightened neuronal activity rather than altered changes in synaptic transmission. Together, these electrophysiological changes suggest functional increased activity of DRD1-MSNs in the dorsal striatum of *Cry1Δ11* mice.

### Aberrant calcium activity in dorsolateral striatal DRD1-MSNs during repetitive behaviors in *Cry1Δ11* mice

**Fig. 4:**
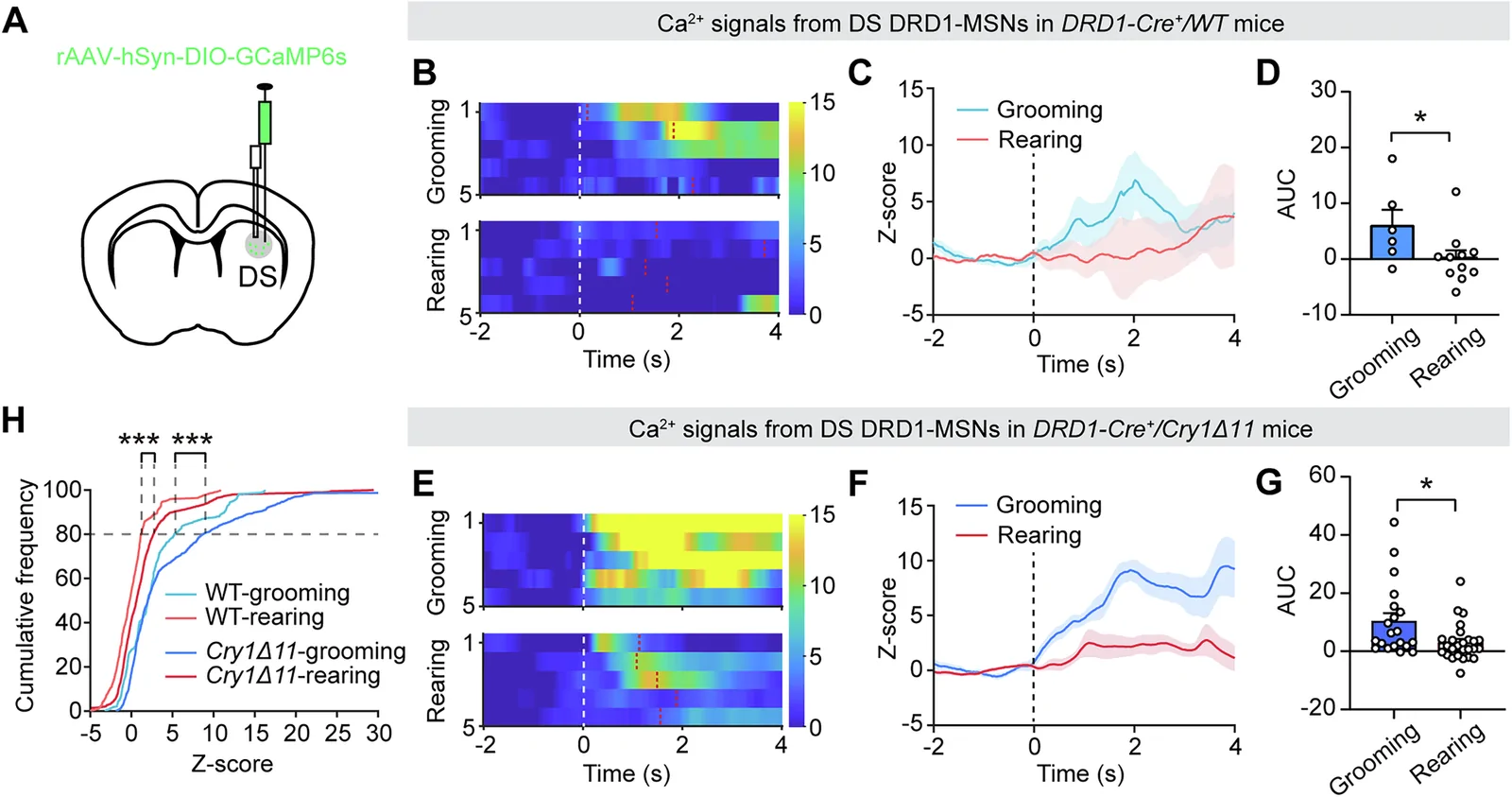
Increased Ca^2+^ signals in DLS DRD1-MSNs of *Cry1Δ11* mice during repetitive behaviors. **A** Schematic diagram illustrating virus injection and fiber photometric canula placement in the right DLS. **B** Heatmaps showing representative Ca^2+^ signals from DLS DRD1-MSNs before and after the onset (white dotted line) of grooming (top) and rearing (bottom) trials in *DRD1-Cre*^*+*^*/WT* mice. Red dashed lines indicate behavioral offsets within the 4-s window; traces lacking a red offset line indicate that the behavior extended beyond this window. **C** Group trace plots of Ca^2+^ signals during grooming and rearing in *DRD1-Cre*^*+*^*/WT* mice. Traces are color coded. **D** Comparison of area under the curve (AUC) for Ca^2+^ signals (Z-score) after the onset of grooming and rearing in *DRD1-Cre*^*+*^*/WT* mice. Each circle represents one trial. n = 3 mice. *P < 0.05, Mann-Whitney *U* tests. **E–G** The same as shown in **(B–D)** but for *DRD1-Cre*^*+*^*/Cry1Δ11* mice. Traces are color coded. n = 3 mice. *P < 0.05, Mann-Whitney *U* tests. **H** Cumulative frequency distribution of Ca^2+^ signals for *DRD1-Cre*^*+*^*/WT* and *DRD1-Cre*^*+*^*/Cry1Δ11* mice during grooming and rearing. Traces are color coded. ***P < 0.001, two-sample Kolmogorov-Smirnov (KS) test. Data are presented as mean ± SEM.

To investigate whether DRD1-MSNs in the dorsal striatum are functionally engaged during repetitive behaviors, we performed in vivo fiber photometry to monitor calcium dynamics. We crossed *Cry1Δ11* mice with *DRD1-Cre* mice and unilaterally injected a Cre-dependent AAV vector (rAAV-hSyn-DIO-GCaMP6s-WPREs) into the dorsolateral striatum (DLS), enabling selective expression of calcium indicator GCaMP6s in DRD1-MSNs (Fig. 4A). After three weeks of viral expression, calcium signals were recorded during spontaneous self-grooming and rearing episodes.

In both genotypes, DRD1-MSNs in the DLS exhibited robust calcium signals (quantified as Z-scores; see Methods) during self-grooming, but minimal activity during rearing episodes (Fig. 4 B–G). Quantification of the area under the curve (AUC) confirmed that calcium signals during grooming were significantly higher than those during rearing in both *DRD1-Cre*^*+*^*/WT* (Fig. 4D) and *DRD1-Cre*^*+*^*/Cry1Δ11* mice (Fig. 4G). Notably, *DRD1-Cre*^*+*^*/Cry1Δ11* mice displayed a greater proportion of large-magnitude calcium events, as indicated by a rightward shift in the cumulative distribution of Z-score during both behaviors (Fig. 4H). Overall, these findings indicate that DLS DRD1-MSNs are preferentially recruited during self-grooming and that their activity is pathologically amplified in *Cry1Δ11* mice.

### Activation of dorsolateral striatal DRD1-MSNs is both necessary and sufficient to drive self-grooming behavior

**Fig. 5:**
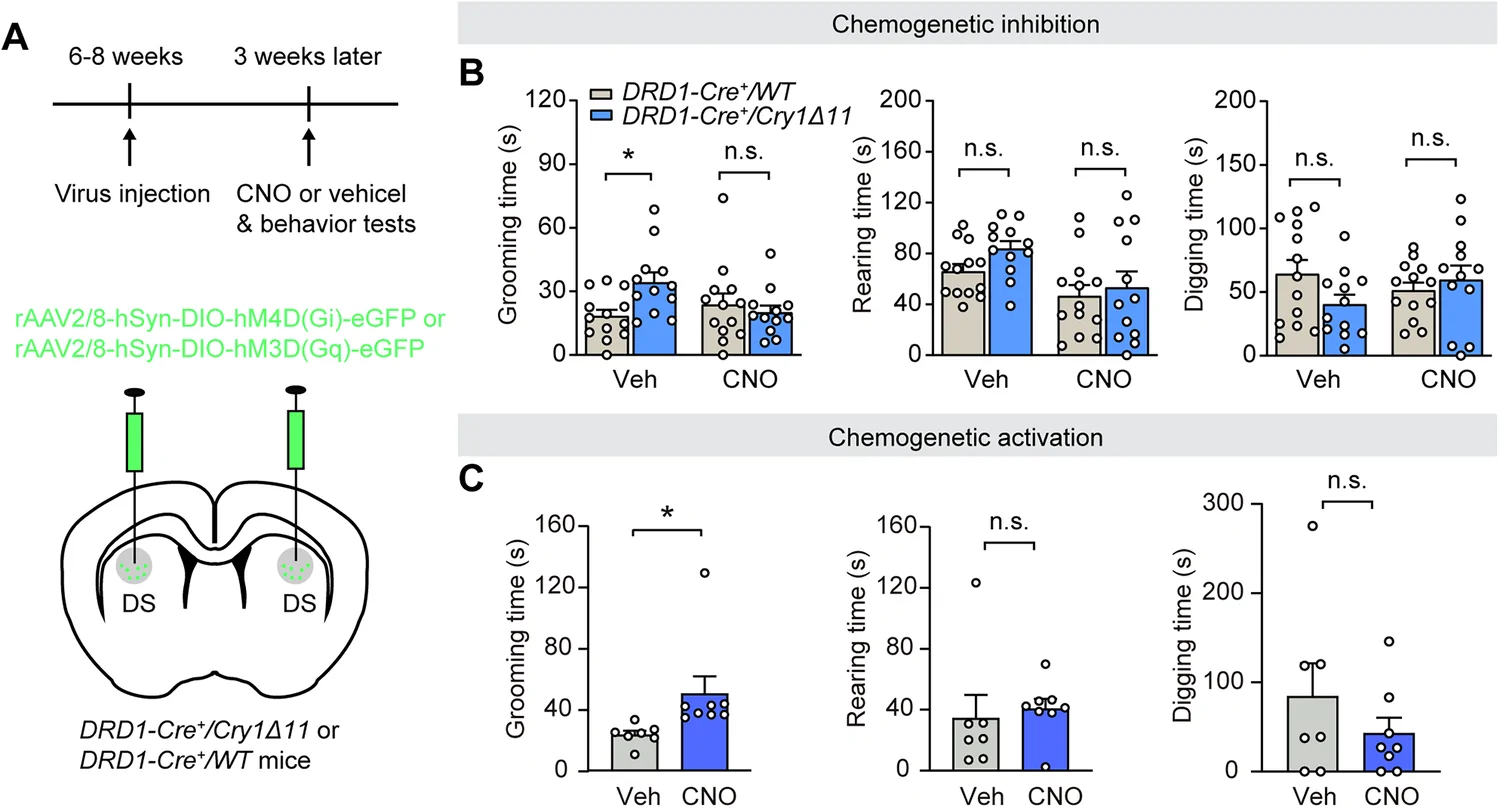
Bidirectional control of self-grooming via chemogenetic manipulations of DLS DRD1-MSNs. **A** Schematic diagram illustrating bilateral viral injections into DLS and the experimental timeline for chemogenetic manipulations. **B** Quantification of grooming, rearing, and digging behaviors in *DRD1-Cre*^*+*^*/WT* and *DRD1-Cre*^*+*^*/Cry1Δ11* mice following intraperitoneal injection of vehicle (Saline, Veh) or CNO in the chemogenetic inhibition experiment. n = 13 *DRD1-Cre*^*+*^*/WT* versus 12 *DRD1-Cre*^*+*^*/Cry1Δ11* mice. *P < 0.05, ns, not significant (P ≥ 0.05), two-way ANOVA followed by Bonferroni post hoc test. **C** As shown in **(B)** but for chemogenetic activation in *DRD1-Cre*^*+*^*/WT* mice. n = 7 mice for vehicle versus 8 mice for CNO condition. *P < 0.05, ns, not significant (P ≥ 0.05), two-tailed unpaired Student’s *t* test. Data are presented as mean ± SEM.

To establish a causal role for DLS DRD1-MSNs in repetitive behaviors, we employed chemogenetic silencing. An inhibitory designer receptor exclusively activated by designer drugs (DREADDs) was selectively expressed in DLS DRD1-MSNs of *DRD1-Cre*^*+*^*/Cry1Δ11* and *DRD1-Cre*^*+*^*/WT* mice via bilateral infusion of rAAV2/8-hSyn-DIO-hM4D(Gi)-eGFP (Fig. 5A). Three weeks later, mice received clozapine N-oxide (CNO; 1 mg/kg) or vehicle (Saline) via intraperitoneal (i.p.) injection 30 min before behavioral testing. CNO administration did not affect behavior in control mice lacking DREADD expression (Supplementary Fig. 3). Chemogenetic inhibition of DLS DRD1-MSNs significantly reduced excessive grooming in *DRD1-Cre*^*+*^*/Cry1Δ11* mice, normalizing their behavior to levels comparable to WT controls, whereas rearing and digging behaviors remained unaffected (Fig. 5B). These results demonstrate that hyperactivity of DLS DRD1-MSNs is necessary for the expression of pathological self-grooming in this model.

To further test sufficiency, we activated DLS DRD1-MSNs in *DRD1-Cre*^*+*^*/WT* mice using the excitatory DREADD system (rAAV-hSyn-DIO-hM3D(Gq)-eGFP; Fig. 5A). Following CNO administration (1 mg/kg, i.p.), WT mice exhibited a significant increase in self-grooming duration compared with vehicle-treated controls (Fig. 5C). In contrast, rearing and digging behaviors were unaffected by DRD1-MSN activation (Fig. 5C). This finding indicates that selective excitation of DLS DRD1-MSNs is sufficient to drive a core repetitive behavior like self-grooming, but may not be the primary driver of other stereotyped actions such as rearing and digging, suggesting distinct neural substrates or thresholds for different repetitive actions.

### Pharmacological inhibition of DRD1 signaling rescues the repetitive behaviors in *Cry1Δ11* mice

**Fig. 6:**
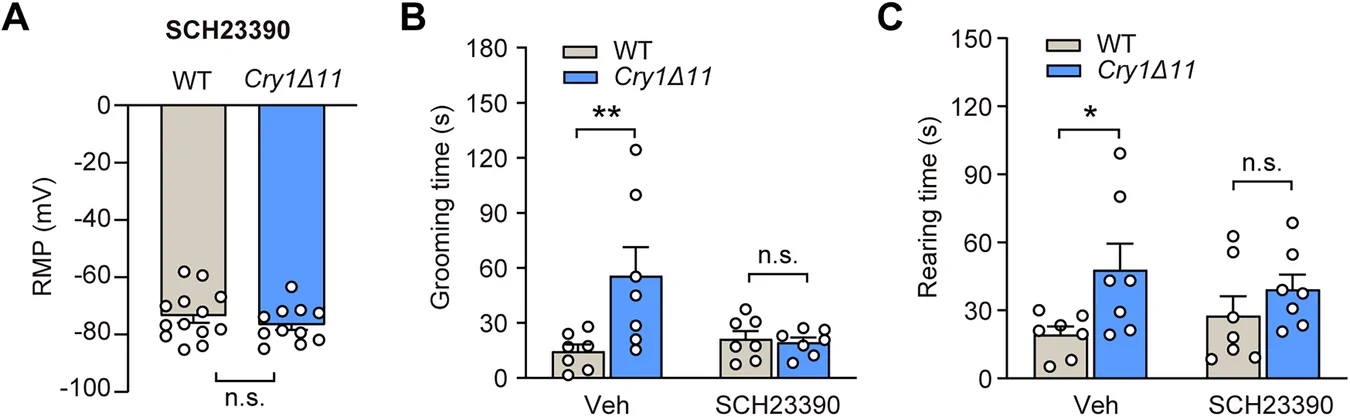
DRD1 antagonist SCH23390 rescues the RMP and repetitive behaviors in *Cry1Δ11* mice. **A** RMP of DRD1-MSNs in WT and *Cry1Δ11* mice treated with the DRD1 antagonist SCH23390 (10 μM). n = 13 neurons from 3 WT mice versus 11 neurons from 3 *Cry1Δ11* mice. ns, not significant (P ≥ 0.05), two-tailed unpaired Student’s *t* test. **B**, **C** Behavioral analysis following intraperitoneal injection of vehicle or SCH23390 (15 μg/kg). Behaviors were measured 30 min post-injection. SCH23390 normalized the hyperactivity in the grooming **(B)** and rearing **(C)**. Vehicle, n = 7 WT versus 7 *Cry1Δ11* mice; SCH23390, n = 7 WT versus 7 *Cry1Δ11* mice. *P < 0.05, **P < 0.01, ns, not significant (P ≥ 0.05), two-way ANOVA followed by Bonferroni post hoc test. Data are presented as mean ± SEM.

Based on the above results, the hyperactivation of DLS DRD1-MSNs in *Cry1Δ11* mice might be due to elevated DRD1 signaling, thus we tested whether suppressing DRD1 activity could rescue these phenotypes. Bath application of the DRD1 antagonist SCH23390 (10 μM) during slice recordings eliminated the RMP difference between two genotypes, restoring the membrane potential of DRD1-MSNs in *Cry1Δ11* mice to WT levels (Fig. 6A). Furthermore, acute systemic administration of SCH23390 (15 μg/kg, i.p.) 30 min before testing fully normalized excessive grooming and rearing in *Cry1Δ11* mice (Fig. 6B, C). This demonstrates that both neuronal hyperactivity and repetitive behaviors are directly dependent on excessive DRD1 signaling.

## Discussion

Our study demonstrates that *Cry1Δ11* mice exhibit robust repetitive behaviors, specifically excessive self-grooming and rearing. Using multimodal approach including immunofluorescence staining, western blotting, in vitro electrophysiology and in vivo fiber photometry, we identified hyperactivity of DRD1-MSNs in DLS as a critical neural substrate underlying these behaviors. Chemogenetic inhibition or activation of DLS DRD1-MSNs bidirectional modulated self-grooming behavior, while pharmacological blockade of DRD1 signaling with SCH23390 effectively rescued both neuronal hyperactivity and behavioral abnormalities in *Cry1Δ11* mice. Together, these findings establish the necessity and sufficiency of DLS DRD1-MSNs activity for the expression of excessive self-grooming, positioning DRD1 inhibition as a promising therapeutic strategy for repetitive behavior in ADHD and related neurodevelopmental disorders.

Growing evidence links circadian clock gene dysfunction to neuropsychiatric disorders [27–29]. Among core clock components, CRY1 has garnered particular interest due to its central role in the transcriptional-translational feedback loop governing circadian rhythms [13]. The *CRY1Δ11* splice variant, a mutation first identified in families with DSPD [14], has been associated with elevated rates of ADHD [15]. In line with prior work from our group, *Cry1Δ11* mice recapitulate multiple ADHD-like phenotypes, including hyperactivity, impulsivity, and cognitive deficits [16] and also exhibit altered pain sensitivity mediated by the spinal cord [17]. Extending these findings, we now show that the same *Cry1Δ11* mutation also drives repetitive behaviors, including excessive self-grooming and stereotyped rearing. Importantly, these repetitive behaviors are not secondary to general hyperactivity. In fact, when we crossed *Cry1Δ11* mice with *DRD1-Cre* lines for cell-type-specific manipulations, the baseline hyperactivity phenotype previously reported [16] was attenuated (Supplementary fig. 4), likely due to *Cre*-mediated disruption of endogenous *DRD1* expression or cytotoxic effects commonly associated with CRE recombinase insertion [30–32]. Similarly, the elevated self-grooming duration observed in *Cry1Δ11* mice (Fig. 1B) was reduced in this cross (Fig. 5B). Nevertheless, the core repetitive phenotype persisted, supporting the specificity of this phenotype.

These findings raise an intriguing question about how a single mutation in a core circadian gene can give rise to multiple distinct neuropsychiatric phenotypes. We propose that *CRY1Δ11* mutation exerts two key effects. First, a recent single-cell atlas revealed robust circadian rhythmicity of c-Fos across multiple brain regions [33]. As a global regulator of the molecular clock, CRY1 may orchestrate circadian transcription throughout the brain. The *CRY1Δ11* mutation may therefore abolish rhythmic c-Fos oscillation in DLS DRD1-MSNs, leading to sustained neuronal hyperactivity. Second, independent of its transcriptional role, CRY1 directly binds Gαs to suppress DRD1 signaling [18], whereas the *CRY1Δ11* mutation disrupts this interaction [16], leading to elevated cAMP/PKA activity and increased c-Fos expression specifically in DRD1-MSNs. Although we did not directly assess the *CRY1Δ11*-Gαs interaction in the present study, DRD1 is the predominant Gαs-coupled GPCR expressed in DLS DRD1-MSNs and is therefore likely the primary driver of the observed hyperactive downstream signaling. Together, these studies suggest that CRY1 functions both as a master transcriptional regulator of circadian rhythms and as a local modulator of dopaminergic signaling in striatal circuits, with dysregulated activity in DRD1-MSNs potentially serving as a shared pathophysiological hub. Whether these roles operate independently or are mechanistically interconnected remains an open question warranting further investigation. Notably, DRD1 is also expressed in the suprachiasmatic nucleus (SCN) [34] and ventral tegmental area (VTA) [35]. Hyperactive DRD1 signaling in these regions could further amplify DLS dysfunction via direct dopaminergic projections or polysynaptic pathways, potentially linking altered striatal activity to broader circadian and behavioral phenotypes.

For repetitive behaviors, animal model studies have identified a distributed network of involved brain regions, including components of the motor control system such as the basal ganglia and cerebellum, limbic structures such as the amygdala and hypothalamus, and the neocortex, which provides top-down regulation [20, 36–39]. Among these, the dorsal striatum has received particular attention as the primary input nucleus of the basal ganglia [40–42]. However, the distinct contributions of DRD1- versus DRD2- MSNs remain debated. Early evidence emphasized a central role for DRD1-MSNs. The DRD1 agonist SKF38393 induces dose-dependent self-grooming in WT rodents [43], and DRD1 antagonism with SCH23390 ameliorates repetitive behaviors in multiple animal models [20, 44], where DRD1-MSNs often exhibit morphological and functional alterations [38, 45]. More recently, DRD2-MSNs have also been implicated, as their chemogenetic or optogenetic [41, 42] inhibition reduces excessive grooming in OCD- and ASD-related models [19]. Rather than reflecting the absolute activity of either population alone, pathological repetition is now considered to result from an imbalance between the direct (DRD1-mediated) and indirect (DRD2-mediated) pathways, a bias that can favor one pathway over the other depending on genetic backgrounds, behavior assays, or brain subregions [37, 42, 46]. In this work, we provide the first evidence that DRD1-MSNs in the DLS are hyperactive during self-grooming in a novel, human-relevant model of ADHD, thereby driving an imbalance that biases striatal output toward the direct pathway.

Repetitive behaviors in rodents are highly diverse and include self-grooming, rearing, digging, jumping, circling, and marble burying [47]. However, only a subset of these behaviors typically manifests in any given animal model, and certain actions may even compete [41, 48]. For instance, *Sapap3*-knockout mice show increased grooming but reduced rearing and locomotion in the open field [41]. Similarly, *Neuroligin*-1 knockdown in DRD2-MSNs leads to their hyperactivity and enhances both grooming and digging, yet chemogenetic activation of these neurons selectively induces grooming without affecting digging [19]. In contrast, *Cry1Δ11* mice in our study exhibit increased grooming and rearing, with no change in digging behavior. Fiber photometry recordings revealed significantly larger calcium transients in DLS DRD1-MSNs during grooming compared to rearing. Moreover, chemogenetic activation of these neurons induced excessive grooming but not rearing. Surprisingly, however, both behaviors in *Cry1Δ11* mice were effectively suppressed by systemic DRD1 blockade. This suggests that while self-grooming is directly driven by DLS DRD1-MSN activity, rearing in this model may be indirectly facilitated through network-level modulation of these neurons. Supporting this interpretation, DLS DRD1-MSNs in *Cry1Δ11* mice exhibited elevated calcium signals during rearing relative to WT controls, indicating heightened engagement of these neurons. Together, these findings suggest that distinct repetitive actions may rely on shared striatal substrate but differ in their dependency on direct neuronal activation versus network-level recruitment.

At the network level, multiple afferent and efferent pathways contribute to repetitive behaviors [20, 36]. First, corticostriatal glutamatergic projections are frequently dysregulated in ASD and OCD models [37, 49, 50]. However, our electrophysiological data reveal no significant difference in synaptic input onto DLS DRD1-MSNs, suggesting that the observed increases in neuronal activity in *Cry1Δ11* mice arises not from altered synaptic input but from intrinsic hyperexcitability. Second, midbrain dopaminergic inputs potently modulate repetitive actions. Recent work identified DRD3-positive granule cells (DRD3-GCs) in the ventral striatal islands of Calleja (IC) as key initiators of grooming syntax [48], which subsequently disinhibit midbrain dopamine neurons [51], potentially amplifying dopamine release in the DLS. Given that DRD3-GCs show ~80% co-expression of DRD1 [52, 53], and that the anterior DLS has been previously implicated in grooming syntax [40], we hypothesize that DRD3-GCs in *Cry1Δ11* mice may also exhibit elevated activity due to DRD1 hyperactivation, thereby reinforcing self-grooming. Third, the functional heterogeneity of the striatum (across dorsal-ventral axes, striosome-matrix compartments, and distinct projection targets) challenges simplistic DRD1/DRD2 dichotomies. Notably, over 30% of DRD1-MSNs project to indirect pathway nuclei [42], challenging classical boundaries. Future studies should therefore adopt circuit-specific, rather than solely molecular, approaches to dissect neuronal function.

In conclusion, our study provides a unified mechanistic framework linking a human-relevant circadian mutation to dopaminergic dysregulation in the DLS and the emergence of repetitive behaviors. Critically, these *Cry1Δ11*-driven behaviors are fully reversible through selective DRD1 antagonism. These findings not only advance our neurobiological understanding of repetitive behaviors but also highlight DRD1 blockade as a potential therapeutic strategy. Given the shared involvement of striatal circuits across multiple neurodevelopmental and psychiatric disorders such as ASD, OCD, and ADHD, this strategy may have transdiagnostic potential for conditions characterized by pathological repetition.

## Materials and methods

### Animals

*Cry1Δ11* knock-in mice were generated using CRISPR/Cas9-mediated genome editing as previously described [16]. DRD1-Cre transgenic mice [54] (EY266line, RRID: MMRRC_034259-UCD) were generously provided by Yousheng Shu and crossed with *Cry1Δ11* mice to generate *DRD1-Cre*^*+*^*/Cry1Δ11* animals. Animals were randomly assigned to control or experimental groups. All experiments used male transgenic mice and their wild-type littermate controls aged 8–12 weeks. Mice were group-housed (4-5 per cage) under standard laboratory conditions (20 ± 2 °C, 50–60% humidity, 12-hour light-dark cycle with lights on at 7:00 AM) with food and water available *ad libitum*. All experimental procedures were approved by the Institutional Animal Care and Use Committee of Central South University.

### Behavioral tests

Mice were handled daily by the same experimenter for at least three consecutive days prior to behavioral testing to minimize handling stress. On the day of testing, animals were habituated to the testing room for 30–60 min. All tests were conducted during the same circadian window (ZT14-16). Repetitive behaviors were assessed in a transparent cylinder (20 cm diameter, 30 cm height) containing a 1-cm layer of standard bedding. After a 10-min habituation period, behavior was video-recorded for 10 min. Between trials, the apparatus was thoroughly cleaned with 30% ethanol to eliminate residual olfactory cues. Repetitive behaviors including self-grooming, rearing and digging were manually scored offline by experimenters who were blind to genotype and treatment conditions. Individual bouts were defined as continuous actions separated by ≥3 s of inactivity. Open-field test (OFT) was conducted in the arena (72 × 72 × 36 cm) and video recorded for 10 min. Total distance traveled were calculated. For pharmacological experiments, the DRD1 antagonist SCH23390 (15 μg/kg; Selleck) or the DREADD activator CNO (1 mg/kg; Selleck) was dissolved in sterile saline and administered via intraperitoneal injection 30 min before testing.

### Western blot

Tissue samples were lysed in 2× SDS lysis buffer (2% SDS, 63 mM Tris-HCl, and 10% glycerol). Proteins in lysates were separated by SDS-PAGE, transferred to PVDF membranes, blocked with 5% non-fat milk in PBS with 0.5% Tween 20, followed by incubation with primary antibodies overnight at 4 °C. Antibodies include c-Fos (Synaptic Systems, P12841), CREB (Cell Signaling Technolofy, 9197), P-CREB (Cell Signaling Technolofy, 9198), β-ACTIN (Cell Signaling Technolofy, 3700). After washing, membranes were incubated with HRP-conjugated secondary antibodies for 1 h. The proteins were visualized using the Pierce ECL Western Blotting Substrate kit (Thermo Fisher Scientific, 32106). Band intensities were quantified by ImageJ (NIH).

### Immunofluorescent staining

Without conducting any behavioral experiments, mice were deeply anesthetized and then transcardially perfused with 0.1 M phosphate-buffered saline (PBS) followed by 4% (w/v) paraformaldehyde (PFA) in PBS. Brains were postfixed in the same 4% PFA solution for 12 h at 4 °C and then in 30% (w/v) sucrose solution for 1–2 days. Coronal sections (40 μm thick) containing the dorsal striatum were collected using a cryostat (Leica TCS SP5). Slices were incubated with primary antibodies c-Fos (diluted 1:400; SYSY, P12841) and DRD1 (diluted 1:400; Sigma-Aldrich, D2944) [26] overnight at 4 °C. The following day, sections were washed three times in PBS and incubated for 1 h at room temperature in the dark with Alexa Fluor-conjugated labeled secondary antibodies. Nuclei were counterstained with DAPI for 10 min. Fluorescent images were acquired using a confocal laser scanning microscope (ZEISS LSM 980) with consistent laser power and gain settings across all experimental groups.

### Stereotaxic surgery and viral injections

Mice were deeply anaesthetized with isoflurane (3% for induction, 1–1.5% for maintenance) and head-fixed in a stereotactic frame (KOPF Instruments, USA). Viral vectors or fluorescent retrograde beads were bilaterally delivered into target brain regions using glass micropipettes connected to a pressure-controlled microinjector (Lianqiweilai, China). Stereotaxic coordinates relative to bregma were as follows: AP, + 1.0 mm; ML, ± 2.4 mm; DV, - 3.5 mm for DLS; AP, - 3.2 mm; ML, ± 1.3 mm; DV, - 4.8 mm for SNr. For fiber photometry experiments, AAV2/9-hSyn-DIO-GCaMP6s-WPREs (titer: 1.0 × 10^13^ vg/mL; diluted 1: 4 in sterile PBS; Taitool, China) was injected into the DLS to label DRD1-MSNs. For chemogenetic manipulation, rAAV2/8-hSyn-DIO-hM3D(Gq)-eGFP-WPRE-pA (titer: 1.0 × 10^13^ vg/mL; diluted 1: 4 in sterile PBS; Taitool, China) or rAAV2/8-hSyn-DIO-hM4D(Gi)-eGFP-WPRE-pA (titer: 1.0 × 10^13^ vg/mL; diluted 1: 4 in sterile PBS; Taitool, China) was used to activate or inhibit the DRD1-MSNs in DLS, respectively. For electrophysiological identification of direct-pathway MSNs, red fluorescent retrobeads (Lumafluor, USA; diluted 1: 4 in sterile PBS) were injected into the SNr. Injection volume was 0.3 μL (for viruses) or 0.1 μL (for retrobeads) per site at a rate of 30 nL/min (for viruses) or 10 nL/min (for retrobeads). The injection pipette was left in place for 10 min post-injection to minimize backflow. For fiber photometry recordings, a 200-μm core diameter optical fiber (NA 0.37; ThinkerTech, China) was implanted 300 μm above the injection site and secured to the skull with dental acrylic (ChangShu ShangChi Dental Materials, China). Following surgery, mice were placed on a heating pad and monitored until fully ambulatory before being returned to their home cages.

### Fiber photometry and analysis

To monitor calcium dynamics in DRD1-MSNs in vivo, GCaMP6s virus was injected into the DLS as described above. Behavior tests were conducted 3–4 weeks after the surgery to allow sufficient viral expression and recovery. During testing, the fluorescence signals were collected using a multi-channel fiber photometry system (ThinkerTech, China) coupled to a 200-μm core diameter optical fiber implanted above the DLS. GCaMP6s was excited by a 470-nm laser with a power of ~30 µW at the tip (tested during constant light). A side-view recording camera (C920 pro, Logitech) was used to assess the behavior of animal in the cylinder. Videos were synchronized with photometry data and later analyzed frame-by-frame for behavioral annotation.

Photometry data were processed offline using MATLAB (R2024a, MathWorks). Raw fluorescence traces (from 470 nm channel) were corrected for photobleaching using a polynomial fit and then subtracted from the isosbestic signal (from 405 nm channel). The corrected signal (F) was then converted to Z-score by calculating ΔF/σF = (F-F0)/σF, where F0 and σF represents the mean and standard deviation of F during a 2-second baseline window immediately preceding behavior onset (e.g., self-grooming initiation). For quantitative analysis, the area under the curve (AUC) of Z-scored calcium signal was calculated over a 2-second window following behavior onset.

### Electrophysiological recording and analysis

Electrophysiological experiments were performed in coronal brain slices containing dorsal striatum. Brain slices (300 μm thick) were prepared from 8- to 12-week-old mice. Mice were deeply anesthetized with sodium pentobarbital (50 mg/kg, i.p.) before decapitation. The brains were rapidly removed and immersed in ice-cold sucrose-based artificial cerebrospinal fluid (ACSF) containing 213 mM sucrose, 2.5 mM KCl, 1.25 mM NaH_2_PO_4_·H_2_O, 26 mM NaHCO_3_, 10 mM dextrose, 2 mM MgSO_4_, 2 mM CaCl_2_. Brain slices were cut using a vibratome (7000 smz-2, Campden Instruments) in this ice-cold ACSF and then transferred immediately to an incubation chamber filled with normal ACSF (126 mM NaCl, 2.5 mM KCl, 26 mM NaHCO_3_, 1.25 mM NaH_2_PO_4_·H_2_O, 25 mM dextrose, 2 mM CaCl_2_, 2 mM MgSO_4_, pH 7.2-7.3, 310–320 mOsm). Slices were maintained at 35.5 °C for 45–60 min and then kept at room temperature until use. During the slicing and recording, solutions were continuously aerated with a mixture of 95% O2 and 5% CO2. Slices were perfused with normal ACSF at 34 °C–35 °C, at a flow rate of 2 mL/min.

Whole-cell recordings were performed under a microscope equipped with infrared differential interference contrast (IR-DIC) optics (BX-51WI, Olympus). Patch pipettes with an impedance of 4–7 MΩ were filled with K-gluconate-based internal solution (for firing properties and mEPSCs) or Cs-based high-Cl internal solution (for mIPSCs). K-gluconate-based internal solution containing (in mM) 140 K-Gluconate, 3 KCl, 2 MgCl_2_·_6_H_2_O, 0.2 EGTA, 10 HEPES, and 2 Na_2_ATP (285–295 mOsm, pH 7.2–7.25). Cs-based high-Cl internal solution containing (in mM) 120 CsCl, 10 TEA-Cl, 1 EGTA, 10 HEPES, 2 QX314-Cl, 4 MgATP, 0.3 Na_3_GTP, and 10 creatine phosphate sodium (285–295 mOsm, pH 7.2–7.25). Recording with series resistance less than 20 MΩ were included for data analysis. Data were acquired using a MultiClamp 700B amplifier (Molecular Devices), Digidata 1550B (Molecular Devices), and pClamp 10.6 software (sampling: 50 kHz; filtering: 10 kHz). The liquid junction potential (~15.6 mV) was not corrected in the data analysis process.

To examine electrophysiological properties such as the frequency-current (F-I) curve and input resistance, positive (50 pA/step, 500 ms duration) or negative (−60 pA, 500 ms) current pulses were applied. The resting membrane potential (RMP) was defined as the cell membrane potential without any current injection. A successful action potential was defined as having an amplitude surpassed 30 mV. For mEPSC recordings, ACSF contained tetrodotoxin (TTX, 1 μM) and picrotoxin (PTX, 50 μM) was used to block the action potential and GABA_a_ receptor, respectively. For mIPSC recordings, ACSF contained tetrodotoxin (TTX, 1 μM) and kynurenic acid (KYN, 1.5 mM) was used to block the action potential and glutamate receptor, respectively. Neurons were voltage-clamped at −70 mV. Continuous recordings of stable mEPSCs and mIPSCs for 60 s were used for data analysis.

### Statistics

Data were analyzed using MATLAB (2024a, MathWorks), MiniAnal 6.0 and Prism 9 (GraphPad Software). All measurements were taken from distinct samples. Normality was assessed by Shapiro-Wilk test. Normally distributed data were compared using two-tailed unpaired Student’s *t* test; non-normal data were analyzed with Mann-Whitney *U* tests and two-sample Kolmogorov-Smirnov (KS) test; data with unequal variances were analyzed with Welch’s t-test. For two-factor designs (e.g., genotype × treatment), a two-way ANOVA followed by Bonferroni-corrected post hoc comparisons was used. Significance levels are denoted as follows: *P < 0.05, **P < 0.01, ***P < 0.001, ns, not significant (P ≥ 0.05). Experimental data in the figures are presented as mean ± SEM.

## Supplementary information

Supplementary figures

## Data Availability

The MATLAB (R2024a, MathWorks) code used in this study is available from the corresponding author (SXD, sxdeng@csu.edu.cn) upon request.
