# Supplementary material for: Increased activity of DRD1-MSNs in dorsolateral striatum underlies Cry1Δ11 mutation-induced repetitive behaviors
Source: Transl Psychiatry. 2026 Jul 13;16:533. doi: 10.1038/s41398-026-04219-8 (PMC13645795; doi:10.1038/s41398-026-04219-8)
Supplement: Supplementary file 1 [file 41398_2026_4219_MOESM1_ESM.pdf]

Supplementary Materials

Increased Activity of DRD1-MSNs in Dorsolateral Striatum Underlies *Cry1Δ11* Mutation-Induced Repetitive Behaviors

Xiran Liu [1,2,3,4,5,6], Dengfeng Liu[1,2,3,4,5,6], Bingyu Long[1,2,3,4,5], Yichun Zhang[1,2,3,4,5], Fan Xia[1,2,3,4,5], Jia-Da Li[1,2,3,4,5], Suixin Deng[1,2,3,4,5*]

[1] Furong Laboratory, Center for Medical Genetics, School of Life Sciences, Central South University, Changsha, Hunan, P. R. China

[2] MOE Key Laboratory of Rare Pediatric Diseases, Changsha, Hunan, P. R. China

[3] Hunan Key Laboratory of Animal Models for Human Diseases, Changsha, Hunan, P. R. China

[4] Hunan Key Laboratory of Medical Genetics, Changsha, Hunan, P. R. China

[5] Hunan International Scientific and Technological Cooperation Base of Animal Models for Human Diseases, Changsha, Hunan, P. R. China

[6] These authors contributed equally

[*] Correspondence: sxdeng@csu.edu.cn

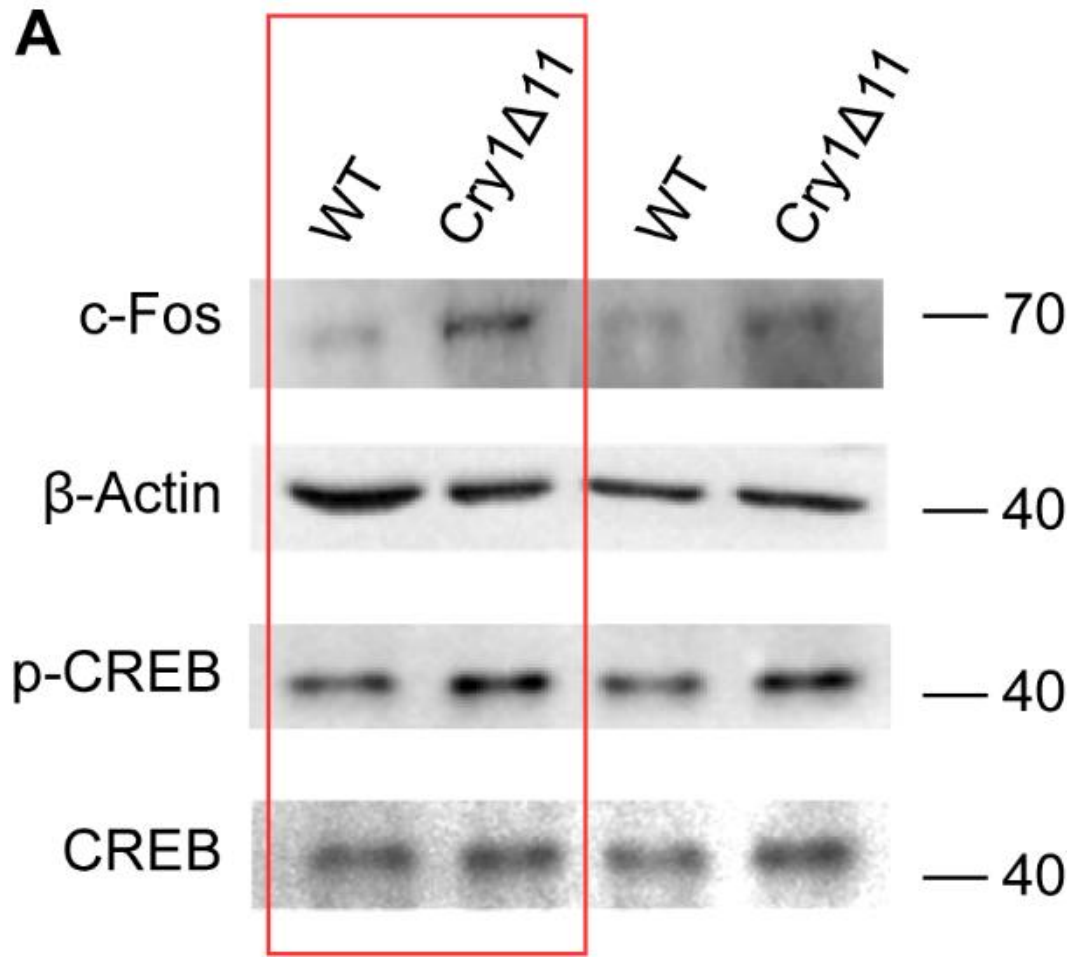

Supplementary figure 1. Full Western blots of c-Fos and CREB.

A Western blots for c-Fos and phosphorylated CREB (p-CREB) from dorsal striatum lysates of four individual mice (two WT and two *Cry1Δ11* biological replicates; lanes 1-2: WT, lanes 3-4: *Cry1Δ11*). The regions within the red boxes correspond to the cropped images presented in the main text.

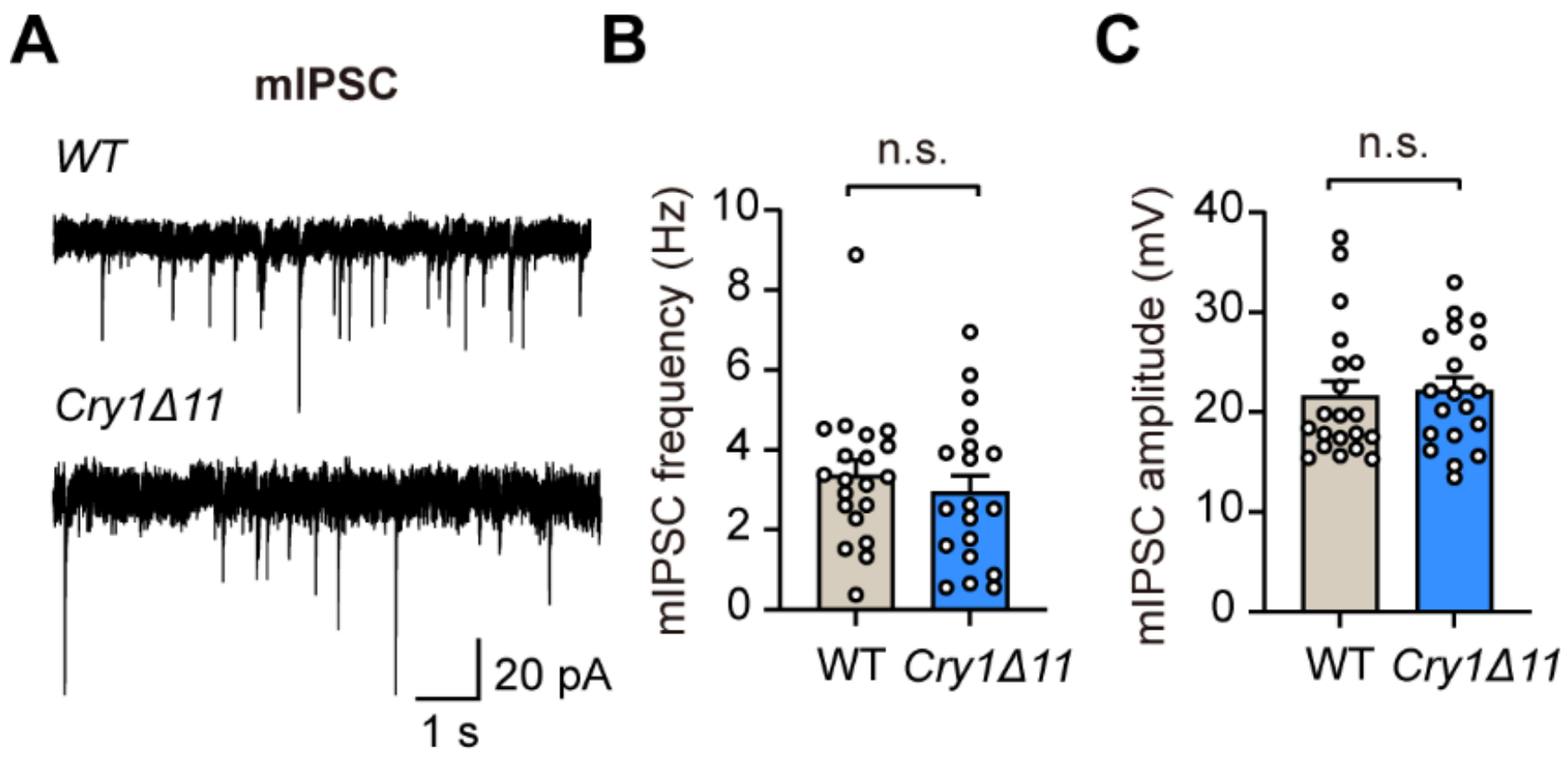

Supplementary figure 2. Dorsal striatal putative DRD1-MSNs exhibit comparable mIPSCs in *WT* and *Cry1Δ11* mice.

A Sample traces of mIPSC recorded from putative DRD1-MSNs in the presence of KYN (1.5 mM) and TTX (1 μM) in WT (top) and *Cry1Δ11* (bottom) mice. **B, C** Quantification of mIPSC frequency **(B)** and amplitude **(C)** from WT and *Cry1Δ11* mice. n = 20 neurons from 4 WT mice versus 19 neurons from 4 *Cry1Δ11* mice. n.s., not significant ($P \geq 0.05$), Mann-Whitney *U* tests. Data are presented as mean ± SEM.

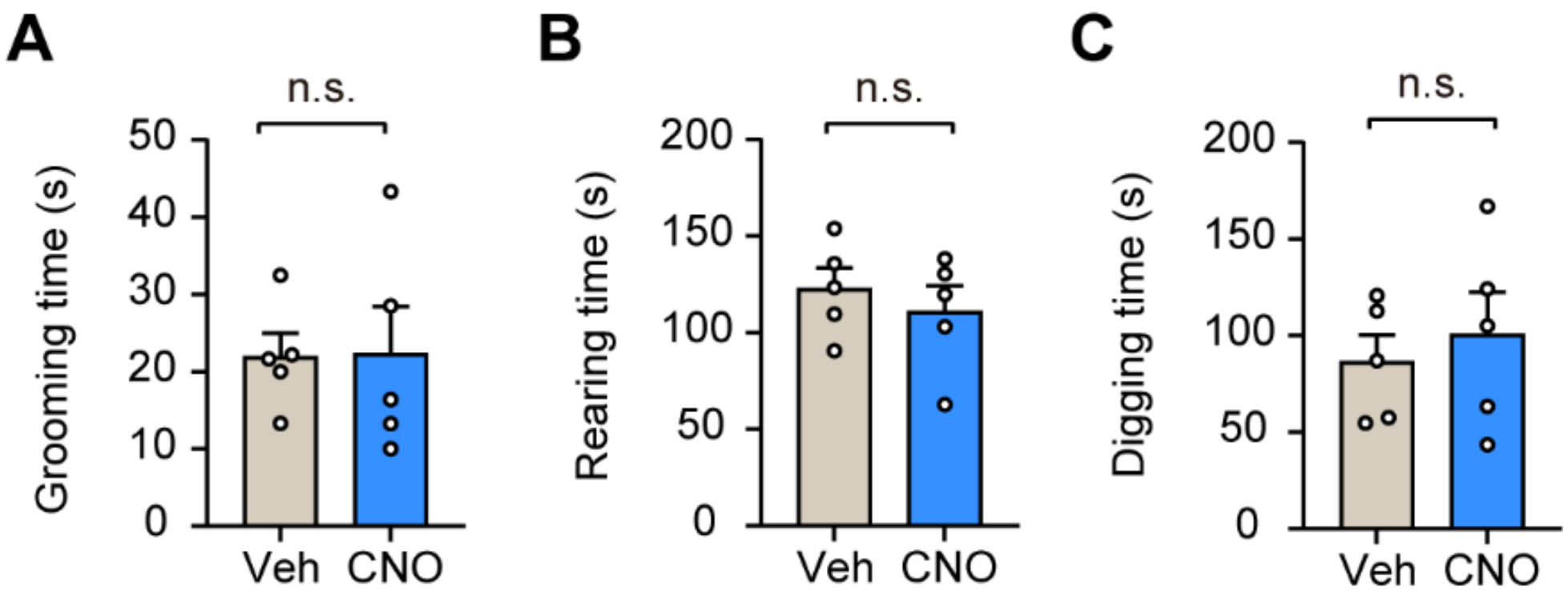

Supplementary figure 3. Intraperitoneal injection of CNO does not significantly alter repetitive behaviors in WT mice.

A-C Time spent grooming **(A)**, rearing **(B)**, and digging **(C)** in WT mice (n = 5) under vehicle versus intraperitoneal CNO conditions. n.s., not significant (P ≥ 0.05), two-tailed unpaired Student's *t* test. Data are presented as mean ± SEM.

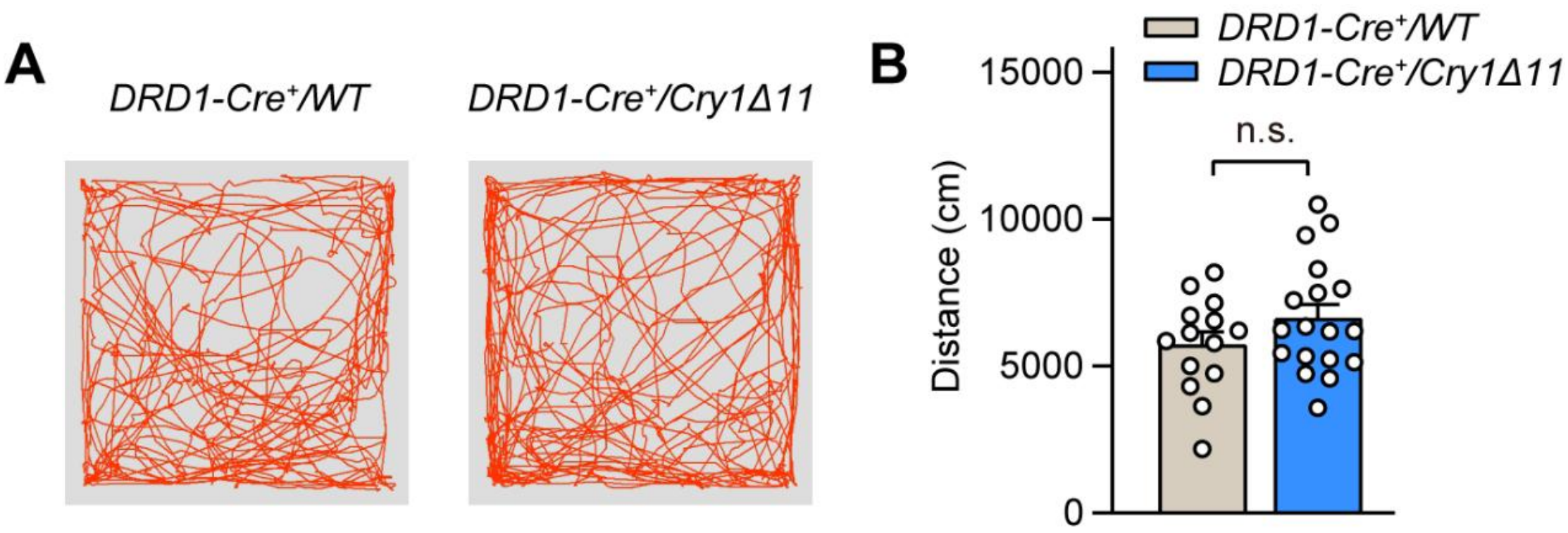

Supplementary figure 4. No hyperactivity is observed in the open-field test of *DRD1-Cre*$^{+}$*/Cry1Δ11* mice.

A Representative movement traces of mice in the OFT. **B** Total distance traveled does not differ significantly between groups. n = 14 *DRD1-Cre*$^{+}$*/WT* versus 18 *DRD1-Cre*$^{+}$*/Cry1Δ11* mice. n.s., not significant ($P \geq 0.05$), two-tailed unpaired Student's *t* test. Data are presented as mean ± SEM.
